# Apoptosis Quantification in Tissue: Development of a Semi-Automatic Protocol and Assessment of Critical Steps of Image Processing

**DOI:** 10.3390/biom11101523

**Published:** 2021-10-15

**Authors:** Juliette de Noiron, Marion Hoareau, Jessie Colin, Isabelle Guénal

**Affiliations:** 1 Laboratoire de Génétique et de Biologie Cellulaire (LGBC), UVSQ, Université Paris-Saclay, 78000 Versailles, France; juliette.bertheault-de-noiron@ens.uvsq.fr (J.d.N.); marion.hoareau@uvsq.fr (M.H.); 2 Ecole Pratique des Hautes Etudes, PSL Research University, 75014 Paris, France

**Keywords:** apoptosis, TUNEL, caspase, image processing, thresholding, signal quantification, *Drosophila*

## Abstract

Apoptosis is associated with numerous phenotypical characteristics, and is thus studied with many tools. In this study, we compared two broadly used apoptotic assays: TUNEL and staining with an antibody targeting the activated form of an effector caspase. To compare them, we developed a protocol based on commonly used tools such as image filtering, z-projection, and thresholding. Even though it is commonly used in image-processing protocols, thresholding remains a recurring problem. Here, we analyzed the impact of processing parameters and readout choice on the accuracy of apoptotic signal quantification. Our results show that TUNEL is quite robust, even if image processing parameters may not always allow to detect subtle differences of the apoptotic rate. On the contrary, images from anti-cleaved caspase staining are more sensitive to handle and necessitate being processed more carefully. We then developed an open-source Fiji macro automatizing most steps of the image processing and quantification protocol. It is noteworthy that the field of application of this macro is wider than apoptosis and it can be used to treat and quantify other kind of images.

## 1. Introduction

Apoptosis is a programmed cell death characterized by caspase activation, subsequent dismantling of cell components, including DNA fragmentation, and final phagocytosis of so called “apoptotic bodies” by surrounding cells or macrophages [1]. Importantly, apoptosis is not only critical for correct development of metazoan organisms, but also for their survival. Indeed, apoptosis failure is observed in many diseases including cancers. Therefore, it is widely studied, and new actors are regularly identified. Apoptosis detection can be performed by multiple methods based on various features of apoptotic steps or regulators. Imaging of apoptosis in whole tissues can rely on a more limited number of methods. The first developed and best known among them is TUNEL (Terminal deoxynucleotidyl transferase dUTP Nick End Labeling) which relies on labeling of DNA 3′ ends whose numbers increase during the DNA fragmentation step of apoptosis. However, TUNEL is costly, time consuming, and also detects necrotic cells [2]. Alternatively, the use of antibodies raised against cleaved—and thus activated—executioner caspases has proved to be more specific and convenient since immunodetection protocols are less time consuming as they include fewer steps than TUNEL. In mammals, the cleaved form of executioner caspase 3 is often targeted [2]. In *Drosophila melanogaster*, the antibody used was raised against the executioner caspase Dcp-1 cleaved at Asp 216. This antibody was recently shown to actually detect the cleaved forms of both Dcp-1 and DrICE executioner caspases [3].

In this study, we wanted to compare TUNEL and cleaved caspase stainings in the most objective way. To this end, we co-stained apoptotic wing imaginal discs (the larval tissue giving the adult wing) with TUNEL and anti-cleaved Dcp-1 antibody and addressed their sensitivity and requirements in terms of image processing.

As a single image can give a wide variety of information, the first step of image analysis consists of choosing a readout (i.e., which data is worth collecting). For example, protein quantity can be assessed by measuring staining intensity, whereas tumor or bacterial colony growth can be followed by measuring the stained area. In the case of apoptosis, the most commonly found readouts are the “number of apoptotic cells” or an “apoptotic index” that has various definitions depending on the lab or experimenters [4,5,6,7].

When the readout is the “number of apoptotic cells”, many studies use a manual counting, implying that an experimenter defines interesting spots and count them. Manual counting is reliable because the expert eyes of experimenters are able to distinguish the signal of interest from background noise better than any machine. This counting can be computer-assisted by software that record every experimenter’s click but even with this assistance, this approach remains time consuming and might involve estimation bias that can potentially raise ethical questions. This is why it is better to rely on automatized—or semi automatized—computer-based methods, whenever it is possible, regardless of the readout. However, machines do not have eyes trained to recognize specific signal from background. This discrimination is allowed by the image processing steps carried out prior to quantification in order to decrease background noise and amplify the signal of interest. This proper discrimination of foreground signal from background noise is called segmentation and defines the boundaries of the objects of interest. Thus, segmentation quality directly affects quantification accuracy.

Software such as Imaris (Bitplane, Belfast, UK) or Matlab (MathWorks, Natick, MA, USA) display default functions for signal quantification [8,9] which usually comprise image processing to yield a rapid result. However, these programs are not open source and the methodology used to obtain the values is often hard to access, meaning that users have only a limited control on their implementation. Moreover, they usually are computationally demanding and thus require powerful devices to run. For all those reasons, many researchers prefer to work with open-source software with which one can develop its own protocol for image processing and quantification. Several open-source image analysis software such as QuPath (University of Edinburgh, Edinburgh, UK) or Icy (Institut Pasteur, Paris, France)have been developed but ImageJ (NIH, Bethesda, MD, USA), and its implemented version Fiji (Fiji Is Just ImageJ, NIH, Bethesda, MD, USA), remain the most widely used as they are highly versatile tools [10,11,12].

Once the readout has been chosen (i.e., intensity, number of objects, area), the image has to be modified to get rid of background noise and artefacts in order to improve segmentation. Images typically display three major kinds of defects: 1. general background noise; 2. isolated background pixels with aberrant high intensities; 3. groups of background pixels with aberrant high intensities. Many functions are available to improve image quality on ImageJ/Fiji software, but they often involve the experimenter’s appraisal. Unfortunately, the more the experimenter is involved, the more it is difficult to ensure that all the images have undergone the same process. However, most of the time, it is impossible to totally exclude experimenter’s involvement.

We previously showed in the *Drosophila* model that overexpressing *rbf1*, the homolog of the human tumor suppressor *RB1*, induces apoptosis in proliferating cells [13]. This apoptosis requires the pro-apoptotic Bcl-2 family member Debcl, and involves caspases activation [14]. It can be visualized using TUNEL on *rbf1* overexpressing wing imaginal discs. In this study, we co-stained wing imaginal discs overexpressing *rbf1* alone (*vg* > *rbf1*) or in the context of a *debcl* partial inactivation (*vg* > *rbf1*, *debcl^E26^*) with both TUNEL and anti-cleaved Dcp-1. We used the images we obtained to compare several methods of image processing and estimate their impact on the quantification of apoptosis for both assays. We then developed a semi-automatic protocol available as a free access Fiji macro called CASQITO (Computer Assisted Signal Quantification Including Threshold Options). This protocol enables to process images from both labelings and quantify the number of apoptotic cells (count readout) or the stained area (area readout). It is worth noting that our analysis and protocol can be relevant to quantify other types of stainings outside the field of apoptosis.

## 2. Materials and Methods

### 2.1. Fly Stocks

Flies were raised at 25 °C on a standard medium. The *UAS-rbf1* and *vg-gal4* strains were generous gifts from Joel Silber (Institut Jacques Monod, Université de Paris, France). The *debcl^E26/E26^* was obtained from the Bloomington Drosophila Stock Center (Bloomington, IN, USA) (BL 27342) and we used a *w^1118^* fly stock as the reference strain.

### 2.2. Immunostaining and Images Acquisition

Third-instar larvae were dissected in 1X PBS pH 7.6 in order to remove all internal tissues except wing imaginal discs, then carcasses were fixed with 3.7% formaldehyde in 1X PBS for 20 min at room temperature and washed three times for 10 min in PBST (1X PBS, 0.3% Triton X-100). Discs still attached to cuticles were then saturated for 1 h in PBST-BSA (1X PBS, 0.3% Triton X-100, 2% BSA) and dissected again to isolate wing imaginal discs which were then incubated overnight with 1:100 dilution of anti-cleaved Dcp-1 (Asp216, Cell Signaling Technology, Danvers, MA, USA) at 4 °C. The following day, after three washes in PBST, wing discs were incubated for two hours with anti-rabbit secondary antibody (1:400, Alexa-Fluor-612-conjugated goat anti-rabbit-IgG (H + L) antibody, Molecular Probes, Thermo Fisher Scientific, Waltham, MA, USA) in PBST. Following three washes in PBST, TUNEL staining was performed according to manufacturer’s instructions (ApopTag Red In situ apoptosis detection kit, merck-millipore, Temecula, CA, USA). Finally, wing discs were mounted in ProLong Diamond (Invitrogen, Waltham, MA, USA) and images were acquired using a Leica SPE inverted confocal microscope (Leica, Wetzlar, Germany) at 568 nm for TUNEL and 612 nm for anti-cleaved Dcp-1 stainings (28 discs for the *vg* > *rbf1* genotype and 39 discs for the *vg* > *rbf1*, *debcl^E26^*). Image analysis was performed exclusively with the Fiji software and the exact same zone was selected for both assays

## 3. Results

### 3.1. Choosing a Readout According to the Biological Question

The readout is the data used to translate the intensity of the biological effect into numbers. Therefore, the chosen readout should be consistent with the biological question and the tool used to study it. For instance, intensity can be measured to assess the amount of a stained component. In the study of apoptosis rate, regardless of the assay, a cell is apoptotic or not. Thus, quantifying the staining intensity, even if it can somehow make sense, does not seem to be the best option for accurate quantification of apoptosis. Conversely, as long as apoptotic cells can be separated from each other (low apoptosis rate, scattered pattern, or intracellular discrete staining), counting the number of objects equates counting the number of apoptotic cells, which constitutes a valid readout. If this readout cannot be used, another valuable readout is the stained area. These count or area measurements can be used as a readout per se or can be used as primary data for calculation of an apoptotic index or score. For instance, such apoptotic index can correspond to a percentage of apoptotic cells obtained by dividing the number of apoptotic cells (count readout) by the total number of cells in the tissue section. As well, another apoptotic index can be obtained from the stained area by dividing it by the cells number (obtained by plasma membrane or nuclear co-staining), by the area of interest (surface of a cellular clone or of the tissue section). These apoptotic indexes do not indicate the actual number of apoptotic cells, which can be estimated using the count readout, but this number of apoptotic cells is rarely necessary, and those readouts provide estimates that are sufficient to compare the apoptosis rate between different samples. Moreover, in a heterogenous tissue, indicating the percentage of apoptotic cells among a specific cell type usually makes more sense.

**Figure 1:**
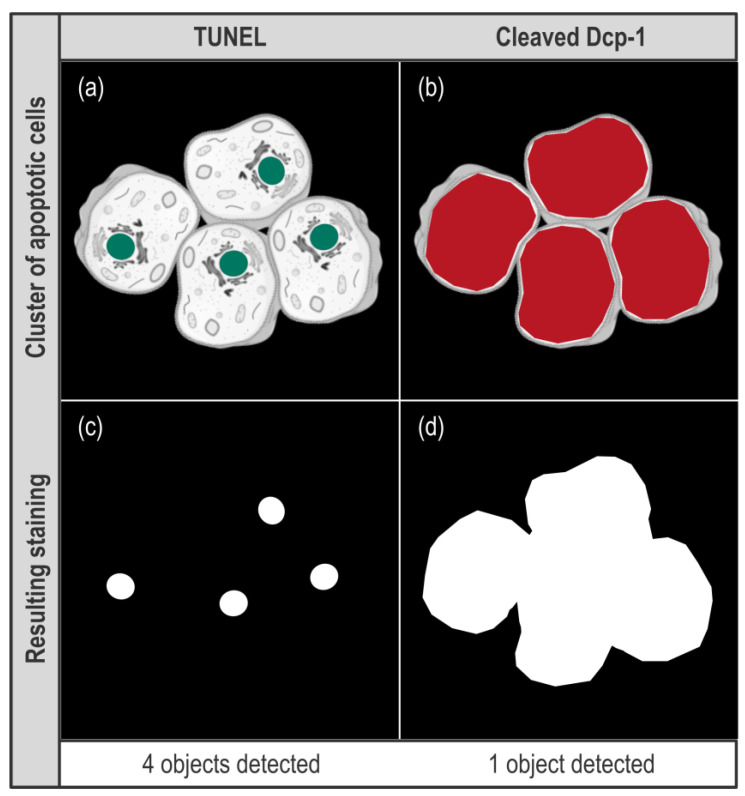
Effect of different types of stainings on the “Count” readout. (**a**,**b**) scheme showing a virtual cluster of four apoptotic cells. In (**a**), green spots represent nuclei stained by TUNEL while in (**b**), red patches represent cytosols stained by anti-cleaved Dcp-1. (**c**,**d**) schemes present the result of image processing for these signals.

Here, we used TUNEL and anti-cleaved Dcp-1 to detect apoptosis. These highlight different features of apoptosis as TUNEL labels fragmented DNA in the nuclei while anti-cleaved Dcp-1 staining is cytosolic. As *rbf1* overexpression is a potent apoptosis inducer in the wing imaginal discs, the probability to have clusters of adjacent apoptotic cells is rather high. This can possibly become problematic for accurate quantification. Indeed, when adjacent cells are apoptotic, TUNEL labeling is expected to remain punctiform as nuclei remain spaced by cytoplasms (Figure 1a,c). On the contrary, with anti-cleaved Dcp-1 staining, it is expected that such adjacent apoptotic cells become indistinguishable from each other and thus appear as a single object (Figure 1b,d).

Therefore, when the readout is the count of apoptotic cells, these clusters of labeled cells are not expected to alter the quantification for TUNEL while they may cause an underestimation of the number of apoptotic cells with anti-cleaved Dcp-1 staining. The extent of this underestimation is difficult to anticipate as it depends on many parameters. Still, this underestimation surely increases with the apoptotic rate—as the probability to have clusters of apoptotic cells increases—which could lead to an artificial flattening of the difference of apoptosis rate that may exist between two conditions. As for the area readout, the size of the wing imaginal disc cells (and their nucleus) being homogenous, the stained area indirectly reflects the number of apoptotic cells without being impacted by their relative localization. In the end, counting cells seems, at least at first sight, a more precise, more direct, readout of apoptosis than area. However, this readout might be altered by apoptotic cell clusters. As it is not possible to anticipate how these clusters will affect the quantification in our experimental set up, we chose to use both count and area readouts. With ImageJ/Fiji, these two readouts can be obtained using the “Analyze Particles” function, which only works on binary 2D images. This means that our image processing protocol should include both z-projection and binarization using a threshold, these two treatments being compatible with our set up. Indeed, as our tissue of interest is a monolayer, z-projection should not affect quantification. Moreover, whether a cell is apoptotic or not, our readouts do not depend on signal intensity and binarization by itself should not affect the quantification.

### 3.2. Designing an Image Processing Protocol

Steps of image processing directly depend on the chosen readout. In order to get both the number of objects and the stained area, our image processing protocol is based on three major steps: 1. Background noise reduction, 2. Compression of our 3D images into 2D by a z-axis projection, 3. Thresholding. Those steps allow appropriate segmentation required for relevant quantification by the “Analyze Particles” function. Importantly, on ImageJ/Fiji, there are many ways to minimize background signal, several ways of compressing a 3D image in 2D and multiple ways to determine a threshold, resulting in countless combinations of possible image processing. In this study, we investigated the weight of these parameters on signal segmentation to end up with an optimized and unbiased protocol for apoptosis quantification.

#### 3.2.1. Median Filter and Size Limitation Efficiently Reduce Artefacts

**Figure 2:**
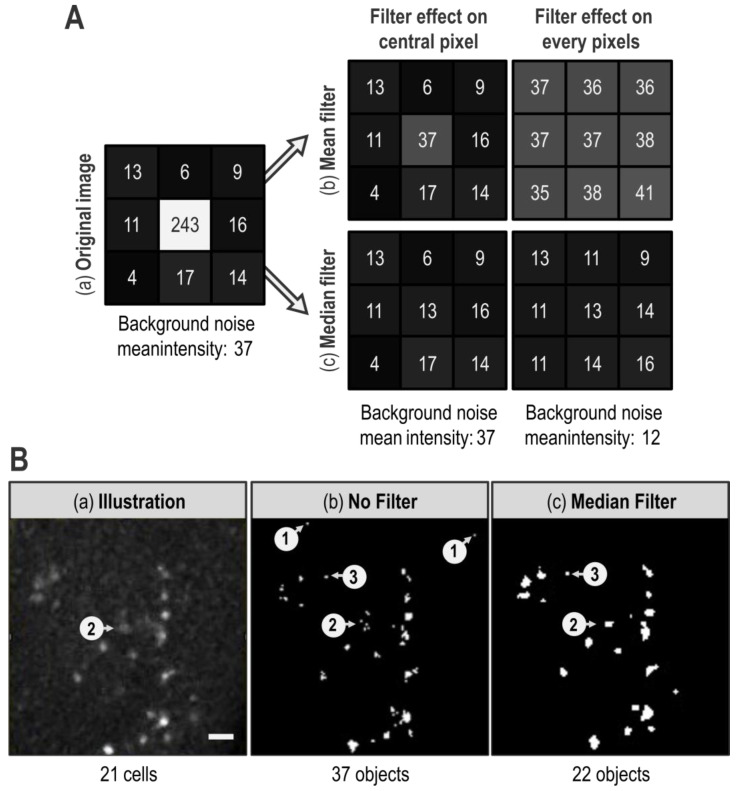
Filters effect on background noise and segmentation. (**A**): The grid in (**a**) presents intensity values of a 3 × 3 pixels image. The grid in (**b**) depicts how a “Mean Filter” with a radius of 1 affects the central pixel of the original image (**left**) and the whole image (**bottom right**). In order to make intensity differences more visible, each boxes background color corresponds to the double of each pixel intensity value in greyscale. (**B**): (**a**) image of a portion of a TUNEL-labeled wing imaginal disc after a Max Intensity z-projection. (**b**) same as (**a**) after binarization using a manually determined threshold. (**c**) same as (**b**) but a “Median Filter” with a radius of 1 was applied to the image before Max Intensity z-projection. White bar corresponds to 10 µm. Arrows with circled numbers 1, 2, and 3 target areas of interest. The number of visible objects in each panel was manually counted.

When quantification is automatized, definition of the signal of interest by segmentation is even more critical. Indeed, wrong segmentation can lead to quantification of unreal objects and thus give useless results. Thus, the signal of interest boundaries has to be better defined while background noise has to be decreased. General background noise can be minimized in many ways depending on the kind of images, the desired readout, and the defaults faced. In our case, mandatory use of a threshold would blacken every low intensity pixel constitutive of the general background noise. However, if binarization of the image efficiently removes diffuse low intensity background noise, it is not sufficient to erase artifactual pixels with aberrant high intensity, i.e., whose intensity is higher than the threshold value. Fortunately, isolated aberrant high pixels can be dealt with filters. Filters are matrix operations that re-calculate a pixel intensity value based on itself and its neighbors. The two mainly used filters are “Mean Filter” and “Median Filter” (Figure 2A).

A “Mean Filter” with a radius of 1 gives a pixel an intensity value that corresponds to the mean of its value and those of its direct neighbors (Figure 2A(b)). Hence, the value of an isolated pixel with aberrant high intensity is attenuated by the intensity value of its neighbors. However, as extreme values impact mean calculation, every neighboring pixel is affected by the isolated aberrant high pixel and their intensity is artificially increased (Figure 2A(b)). By contrast, a “Median Filter” with a radius of 1 gives to a pixel an intensity value corresponding to the median of its value and those of its direct neighbors (Figure 2A(c)), which is expected to be much closer to local intensity value. Besides, as extreme values effect on median calculation is low, high intensity isolated pixel impact on its neighbors is negligible. Overall, the “Mean Filter” tends to spread an aberrant high value whereas a “Median Filter” tends to confine it. This effect is illustrated in Figure 2B, where the “1” arrows of the “No filter” panel show typical isolated aberrant high pixels that are efficiently erased by a “Median Filter” with a radius of 1 (Figure 2B compare (b) and (c)). Aside from this benefit, the area pointed by the “2” arrow exemplifies the median filter ability to preserve edges of an object. Indeed, on the illustration, human eyes easily detect that the “2” arrow targets a marked cell (Figure 2B(a)). However, this object is heterogeneous: in a restricted space, it contains few pixels of high intensity and many pixels of low intensity (i.e., below the chosen threshold). Without any filter, only high intensity pixels are kept after the thresholding step thereby fragmenting this object in several small groups of pixels (Figure 2B(b)). Thus, with no further treatment, multiple objects will be counted in this area, which does not reflect reality. However, as these high intensity pixels are close to each other, the “Median Filter” with a radius of 1 homogenizes intensity values within this object. This allows its reconstruction and gives a segmentation consistent with reality (Figure 2B(c)). Thus, when the “Median Filter” with a radius of 1 is applied, the “number of objects” decreases only to be closer to what a human eye would count.

Although the “Median Filter” with a radius of 1 efficiently reduces the number of artifactual objects by erasing isolated high pixels, the issue of groups of pixels with an intensity higher than the threshold value remains. A way to eliminate most of those artifacts is to limit our analysis to objects with a size consistent with the smallest biological object of interest. In our case, this smallest biological object is a TUNEL-labeled nucleus, we assessed their size on a few random images and thus set a size limit at 2 µm. Importantly, this “>2 µm size limitation” fits our data but should not be taken as a default value and must be adapted for other kinds of signals or cell types. Moreover, as the “Analyze Particles” function records the size of every object, this filtering can be carried out after quantification. In Figure 2B, the “>2 µm limitation” eliminates artifactual objects pointed out by the “3” arrow as well as individual pixels such as those pointed by the “1” arrows. It thus appears very powerful in order to “clean” the image. However, as efficient as the size limitation may be, it cannot replace “Median filter”. Indeed, as already explained, in the absence of a “Median Filter”, the cell indicated by the “2” arrow in Figure 2B becomes fragmented in several small groups of pixels, each one being smaller than 2 µm (Figure 2B(b)). Thus, without the “Median Filter”, these pixels are eliminated by the “>2 µm limitation” and the actually labeled cell indicated by the “2” arrow is not included in the quantification of the apoptotic signal. Here, reconstruction of the object by the “Median Filter” prevents its elimination by the “>2 µm limitation” (Figure 2B(c)). In the end, combination of a “Median Filter” with a radius of 1 and “>2 µm limitation” allows a better segmentation and a more accurate quantification.

#### 3.2.2. Max Intensity Z-Projection Improves Contrast

Confocal microscopy gives the possibility to capture objects in 3D. However, image processing often requires transforming volumes into 2D images by compressing the z-axis. In our case, the “Analyze Particles” function used to quantify the signal of interest requires 2D images. Flattening a 3D volume can seem counterproductive, as separate objects on the same z-axis will be reduced to a single one on the final 2D image. In our case, this is unlikely to happen since imaginal disc cells are organized in a monolayer with only limited folding and this can also be true for tissue sections as long as they are thin enough. In the case of a multilayer tissue, z-projection can still be used if the apoptosis rate is low enough to prevent the occurrence of having multiple apoptotic cells in the same z-axis.

**Figure 3:**
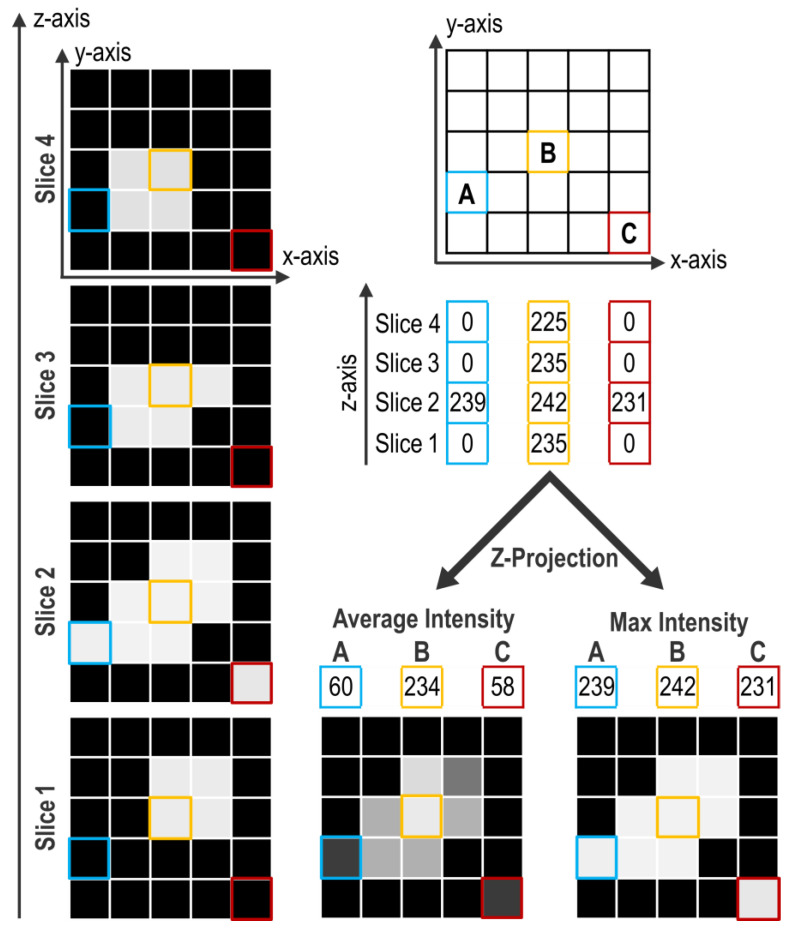
Projection methods. The left panel presents different planes obtained by imaging a virtual object surrounded by a perfect background noise of 0. On the right, the upper panel presents the coordinates of three pixels: A, B, and C, and their respective intensity values along z-axis. Bottom panels show the resulting projection obtained either by an Average Intensity or a Max Intensity projection with respective intensity values obtained for A, B, and C pixels. For all representations, boxes background color corresponds to their pixel intensity in grey scale.

Projection consists of compressing the signal contained in every pixel of a z-axis into a single one. With ImageJ/Fiji, projection can be performed in several ways but two of them are mainly used. The first method, Average Intensity (AI), calculates the average intensity of all the pixels of a z-axis. The second, Max Intensity (MI), only retains the maximal intensity value along the z-axis. Figure 3 presents examples of these projection methods on a virtual object without any other treatment (i.e., median filter).

The z-axis presented in “A” shows only one illuminated pixel on slice #2 and this pixel is included in the object. The “B” z-axis shows numerous pixels highly illuminated, comprised in the object. The “C” z-axis presents an artifactually illuminated pixel on slice #2 that is not comprised in the object. Comparison of the projection methods shows that AI projection decreases the importance of the artifactual pixel of the “C” z-axis, while the MI projection increases its weight. However, in the processing protocol, the preceding use of a “Median Filter” with a radius of 1 eliminates most of those artefacts that are thus not present anymore at the projection step. Conversely, the AI projection of the “A” z-axis leads to loss of signal even if it is part of the object. Furthermore, with the AI projection, the contrast between object and background is weak, therefore the range for the appropriate threshold value is limited (Figure 3). After a MI projection, contrast is enhanced and thus threshold determination is easier for the experimenter, which helps limiting the experimenter bias. This is particularly important for signals with low contrast such as TUNEL. In our case, these two projection methods do not end up in drastically different results but, overall, MI projection presents more benefits than AI projection.

#### 3.2.3. Use of Custom Manual Thresholds Gives the Best Segmentation for Relevant Quantification

The “Analyze Particles” function used for quantification requires the image to be binary. The transition from a greyscale image to a black and white image involves the setting of a threshold that defines an intensity value above which a pixel is turned to white and under which a pixel is turned to black. Ideally, this value should enable to get an image where white only corresponds to the signal of interest. Thresholding is the last step of segmentation and finally defines the objects of interest, which is critical for accurate quantification. Therefore, among the steps of image processing, it is the one that has the most dramatic effect on quantification accuracy, thus we dedicated particular attention to the threshold determination method.

**Figure 4:**
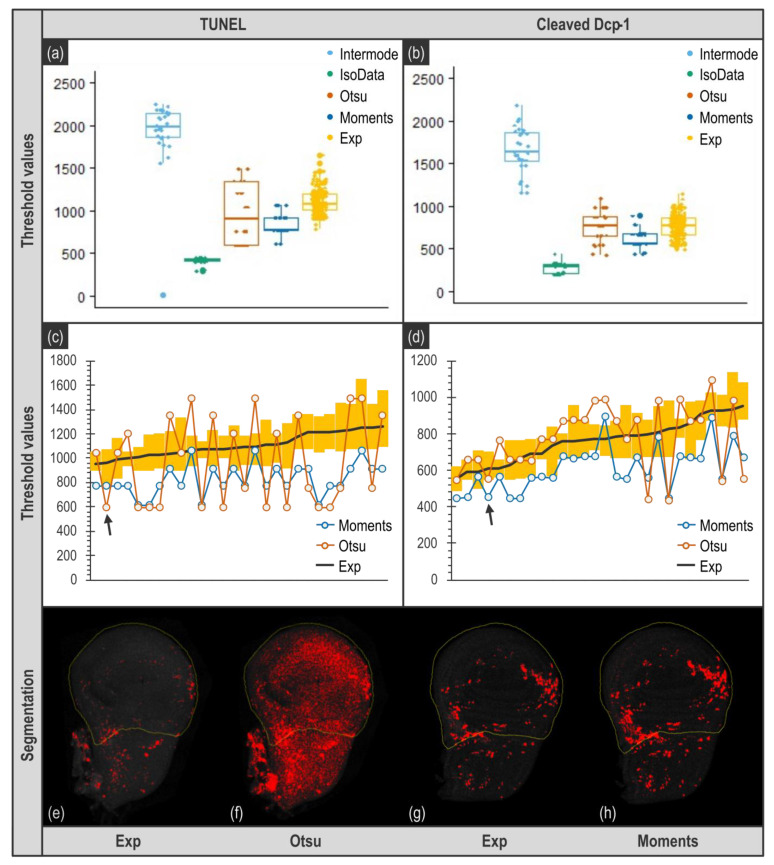
Comparison of thresholding methods. Upper panel presents overall distribution of threshold values per image obtained with various thresholding methods for images of *vg* > *rbf1* genotype. (**a**,**b**) Exp distribution (in yellow) corresponds to the whole of the threshold values determined by three experimenters in triplicate (raw data are presented in Supplementary Figure S1). Intermode (in light blue) and IsoData (in green) are examples of algorithms yielding inadequate values, very far from experimenters’ distribution. Otsu (in brown) and Moments (in blue) are algorithms that seemed usable. (**c**,**d**) show a pairwise organization of the threshold values for each image obtained by Otsu (in brown), Moments (in blue) and experimenters (Exp, the black line corresponds to the mean of the nine values determined the experimenters and the yellow bars correspond to the range of the threshold values determined by the experimenters for each image). Images were ranked according to the mean of the experimenter values (black line). Bottom panel illustrates the result of the binarization using the threshold values determined by experimenters (**e**,**g**), Otsu (**f**) and Moments (**h**). Black arrows of the middle panels target the image used for illustrations presented on the bottom panel. Importantly, these images were chosen as they are representative of the deviation between the algorithm and the experimenter average value (chosen images have a deviation equal to the median of the deviations).

Threshold can be automatically set by algorithms that analyze specific features of the image intensity histogram to determine a threshold value using either simple indicators such as the mean, maximal, or minimal intensity values, or more complex formulas. Hence, algorithms appear as an unbiased method to obtain a specific threshold value per image. We thus wondered whether any of the 16 thresholding algorithms currently available in the menu Image > Adjust > Threshold of ImageJ/Fiji could be used to determine a threshold that properly segments apoptotic signal in our images. Using some randomly chosen images, after application of a “Median filter” with a radius of 1 and a Max Intensity projection, we visually checked if these algorithms could provide a threshold value allowing a relevant segmentation (i.e., consistent with apoptotic signal). Most algorithms did not pass the visual inspection step as they yielded unrealistic segmentation either by ignoring a great portion of the signal or by including artifactual signal. However, two of them, Otsu [15] and Moments [16], seemed capable of discriminating actual apoptotic staining from background. We then performed a more detailed analysis of the threshold values obtained with these algorithms by comparing them to the ones obtained by experimenters. To this end, for the 28 images of the *vg* > *rbf1* genotype, three experimenters determined the threshold to use for each staining (TUNEL or anti-cleaved Dcp-1) by eye and in triplicate (see Supplementary Figure S1). The criteria for manual threshold determination were to set a threshold value that allows the positive signal to be consistent with the original image and located in the vestigial domain. Thresholds for these images were also determined using the 16 algorithms. As expected, algorithms inducing obvious unrealistic segmentation of the apoptotic signal yielded threshold values very far from the range of the ones determined by experimenters (Figure 4a,b; compare IsoData and Intermode with Exp. Data not shown). On the contrary, Moments, Otsu, and experimenter threshold values are in the same range (Figure 4a,b).

From this global analysis, it could seem that algorithms can be as efficient as human eye for threshold determination (compare for instance Otsu and Exp in Figure 4b). However, visually, Otsu capability to determine a relevant threshold seemed irregular. We thus further deepened our data analysis and compared the threshold values obtained not globally but for each image. As shown in Figure 4c,d, the values obtained with Otsu, if they tend to be roughly the same on average than the ones obtained by experimenters, are actually most of the time out of the range of experimenters values. This is particularly striking for images obtained from TUNEL (Figure 4c) as Otsu’s determined values are far higher or lower that the ones obtained by any experimenter. This would not be an issue as long as the threshold values obtained still allow a realistic segmentation of apoptotic signal and subsequent relevant quantification. However, such deviation of the threshold value results in an inappropriate segmentation (compare Figure 4e,f), that necessarily ends in a biased or most likely totally wrong quantification. When it comes to Moments, it provides threshold values that are usually lower than experimenters’ ones (Figure 4a,b), which means that using this algorithm tends to include some background noise to the quantification. The question resides then in determining whether this amount of background noise is important enough to alter quantification. In the case of TUNEL staining (Figure 4c), values are quite low, thus it certainly affects quantification rather significantly. By contrast, when images come from anti-cleaved Dcp-1 staining, the threshold values given by Moments are much closer to the ones obtained by experimenters, they actually seem very similar to the lowest values determined by experimenters (Figure 4d). Therefore, one could assume that the variability of threshold values between Moments and an experimenter is comparable to the one that exists between experimenters. We tested this by comparing the relationship between the two most distant experimenters’ batch of measurements to the one between Moments and its closest dataset. As shown in Supplementary Figure S2B, if two experimenters do not determine exactly the same value for the threshold, their estimates remain consistent with each other (*p* = 10^−5^ and R^2^ = 0.53 for the most distant measurements), the difference can be more or less described as a given experimenter tending to set thresholds always lower than the other. This is a systematic error that should affect quantification only moderately. On the contrary, threshold values determined by Moments are not consistent with the values of experimenters (*p* = 0.8 for the closest in Supplementary Figure S2B). This indicates that Moments can set a low threshold value when all experimenters would have chosen a higher one, but it is not always the case, and most of all, the extent of this underestimation (i.e., the range of the difference between experimenters and Moments’ threshold values) is variable. This is more problematic as the extent of background incorporation in the quantification will then vary and might alter quantification relevance.

Contrary to algorithms, manual determination of the threshold values appears quite robust. Indeed, comparison of manually determined threshold values between experimenters for individual images shows a low variability and a good reproducibility both between several determinations of a given experimenter and between experimenters (Supplementary Figure S1). As all images from an experiment are acquired identically, with the same microscope settings and come from samples treated with the same solutions, at the same time, theoretically, the appropriate threshold value is expected to be the same for every image. Moreover, using the same unique threshold value for all images can be considered as more objective and unbiased.

**Figure 5:**
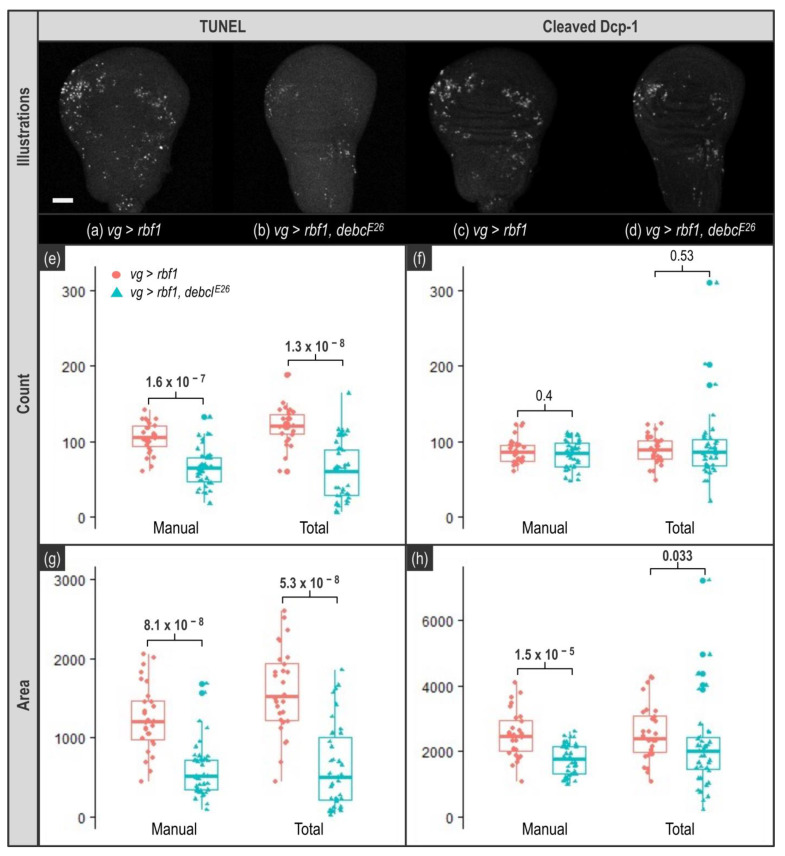
Impact of thresholding on quantification. TUNEL and anti-cleaved Dcp-1 staining quantifications. Upper panel shows representative images of wing imaginal discs stained by TUNEL (**a**,**b**) or anti-cleaved Dcp-1 (**c**,**d**) for *vg* > *rbf1* (**a**,**c**) and *vg* > *rbf1*, *debcl^E26^* (**b**,**d**) genotypes. White bar corresponds to a 50 µm scale. “Count” panel presents quantification of TUNEL (**e**) and anti-cleaved Dcp-1 (**f**) signal based on the counting of the number of objects according to the different thresholding methods (Manual and Total) for *vg* > *rbf1* (in pink) and *vg* > *rbf1*, *debcl^E26^* (in blue). “Area” panel presents quantification of TUNEL (**g**) and anti-cleaved Dcp-1 (**h**) signal based on the number of white pixels (stained area) according to the different thresholding methods (Manual, or Total) for *vg* > *rbf1* (in pink) and *vg* > *rbf1*, *debcl^E26^* (in blue). Manual indicates a custom dedicated threshold was used for each image whereas Total indicates that the same common unique threshold value was used to process all images. This unique threshold value is the median of the median per genotype of the manually determined values. *p*-values displayed above results were obtained using Wilcoxon tests.

However, manually determined threshold values display some variability (Figure 4, Exp). This can justify using a distinct individual, manually determined, threshold value for each image as it might enable a more accurate segmentation and subsequent quantification. In order to assess to which extent these two thresholding methods (unique threshold value for every image or individual, manually determined threshold value per image) can affect quantification and detection of our biological effect, we tried both (Figure 5).

In the Manual condition, each image was binarized using its own manually determined threshold value (determined by experimenter 1, measure 3). From these individual threshold values, we calculated the median value per genotype and then the median of these medians. This latter value was used as the unique threshold value to binarize all images in the Total condition (1128 for TUNEL and 777 for anti-cleaved Dcp-1). We decided to use the median of the medians per genotype rather than the global median (calculated from the whole of the images independently of their genotype) to avoid giving more weight to a genotype (that might have a larger headcount for instance).

For TUNEL stained images, both thresholding approaches give a workable quantification both for count and area readouts (Figure 5e,g). Indeed, a significant decrease in apoptosis between the two genotypes is detected in all cases. However, even if Total or Manual thresholding methods enable to detect the biological effect, we noticed that quantification is still somehow altered when a unique threshold is used (Supplementary Figure S3).

As for anti-cleaved Dcp-1-stained images, the first observation is that the count readout was not usable. Indeed, we knew that apoptotic cells clusters might alter quantification as these clusters might be considered as a single object. Moreover, such underestimation is enhanced when the apoptosis rate rises, eventually leading to the flattening of the difference between two samples. However, we chose to keep this readout, as it was not possible to anticipate the extent of this phenomenon in our samples. As shown in Figure 5f, with this readout, the difference in the apoptosis rate between the two genotypes becomes undetectable. This indicates that the level of apoptosis induced by *rbf1* generates apoptotic cells clusters frequently enough to significantly alter quantification, and this, whatever the thresholding method, thus prohibiting the use of the count readout. By contrast, when anti-cleaved Dcp-1 staining is quantified using the area readout, the difference between the two thresholding methods (unique versus individual thresholds) becomes obvious. As shown in Figure 5h, when a unique threshold value is used for all images (Total), the difference of apoptosis rate between the two genotypes is barely detectable (*p* = 0.033). Moreover, even with an individual, manually determined threshold value per image, all the quantification results show a noticeable variability (Figure 5, Manual) due to differences between specimen, variation of the UAS/gal4 system intensity and small asynchrony in the development of the larvae. However, use of a unique threshold value to process every image tends to increase this variability and induces the appearance of extreme values compatible with an overestimation due to inadequate segmentation (see highest values for *vg* > *rbf1*, *debcl^E26^* genotype in Figure 5h and also Supplementary Figure S3). On the contrary, the use of individual specific threshold values (Manual) enables to readily detect the difference of apoptosis rate between the two genotypes (*p* = 1.5 × 10^−5^).

In the end, this analysis shows that, in our case, using an individual threshold per image is more appropriate and turns out to be the safest option for accurate segmentation, and thus relevant quantification.

### 3.3. Quantifications of TUNEL or Anti-Cleaved Dcp-1 Stainings Do Not Have the Same Requirements

TUNEL and anti-cleaved caspase stainings are widely used to assess the level of apoptosis in tissues. However, depending on the experimental set-up, the quantification step may become delicate.

TUNEL appears as a quite robust apoptosis detection technique. Indeed, it allows to quantify apoptosis and to detect differences in apoptosis rate whatever the thresholding approach, and with both count and area readouts (Figure 5e,g). This was not totally expected since in our images, there was not a strong contrast between the apoptotic signal and the background (Figure 5a,b). However, as previously mentioned, TUNEL assay is costly, time consuming and lacks specificity as it also detects necrotic cells.

On the contrary, using antibodies against cleaved caspase(s) is considered as a more specific and convenient staining of apoptotic cells. By contrast with TUNEL which labels nuclei, caspase staining covers the whole volume of the cell, raising the issue of adjacent apoptotic cells when the readout is counting cells. Indeed, counting the number of apoptotic cells stained with anti-cleaved caspase antibody is perfectly possible as long as apoptotic cells are sufficiently separated from each other. In our experimental set-up, it appeared that the apoptosis rate was too high to prevent the underestimation of the signal due to fusion in a single object of clustered apoptotic cells. When the stained area was measured, it revealed that images from anti-cleaved Dcp-1 stainings should be carefully processed because determination of the threshold value to use for binarization is particularly important. Indeed, even if the difference of apoptotic rate between the two genotypes was known and easily seen by eye (Figure 5, compare (c) and (d)), its detection after quantification was not obvious. Actually, the decrease in apoptosis between the two genotypes is barely detected when a unique threshold value is used for the segmentation of all images (Figure 5h) whereas using a specific threshold value for each image enables to see it. Therefore, anti-cleaved Dcp-1 staining quantification is more affected by image processing than TUNEL and should be handled more carefully.

### 3.4. Macro Explanation

Once the image processing protocol was established and validated, we worked on its automatization. Indeed, doing this processing for every image manually is not only time consuming but also error-prone since it increases the probability to skip or treat twice an image or to make mistakes during data collection. We automatized this image processing protocol by developing an open-source macro called CASQITO (Computer Assisted Signal Quantification Including Threshold Options, available at https://github.com/JdNoiron/CASQITO, accessed on 10 October 2021). Importantly, if the image protocol presented here can be manually performed both with ImageJ and Fiji, the CASQITO macro only runs with Fiji. This macro limits experimenter’s involvement to threshold determination. As we only worked on Leica microscopes, this macro only supports .lif files and should be adapted for other formats. During processing of the images, the macro generates several files for every given .lif file, which will be stored in the same folder as the parental .lif file. We thus recommend recording images from different conditions (genotypes or treatments) in separate .lif projects. The first file generated is a .txt file that recapitulates data from the Log window, which conserves settings associated with the treatment of the .lif file. Two .xls files containing results are also generated, respectively, compiling threshold determination and quantification results. It is worth noting that the latter provides all possible quantifications obtainable with the “Analyze Particles” function whatever the chosen readout may be. A .png file is also generated to display a histogram representing the distribution of the obtained threshold values. If a zone selection is required, another .png file is generated for each image to display experimenter zone selection. Lastly, the macro is not able to process multiple regions of interest on the same image, thus, even if two objects (in our case two wing imaginal discs) are in the same field and can be captured in the same image, it is highly important to capture this field twice.

**Figure 6:**
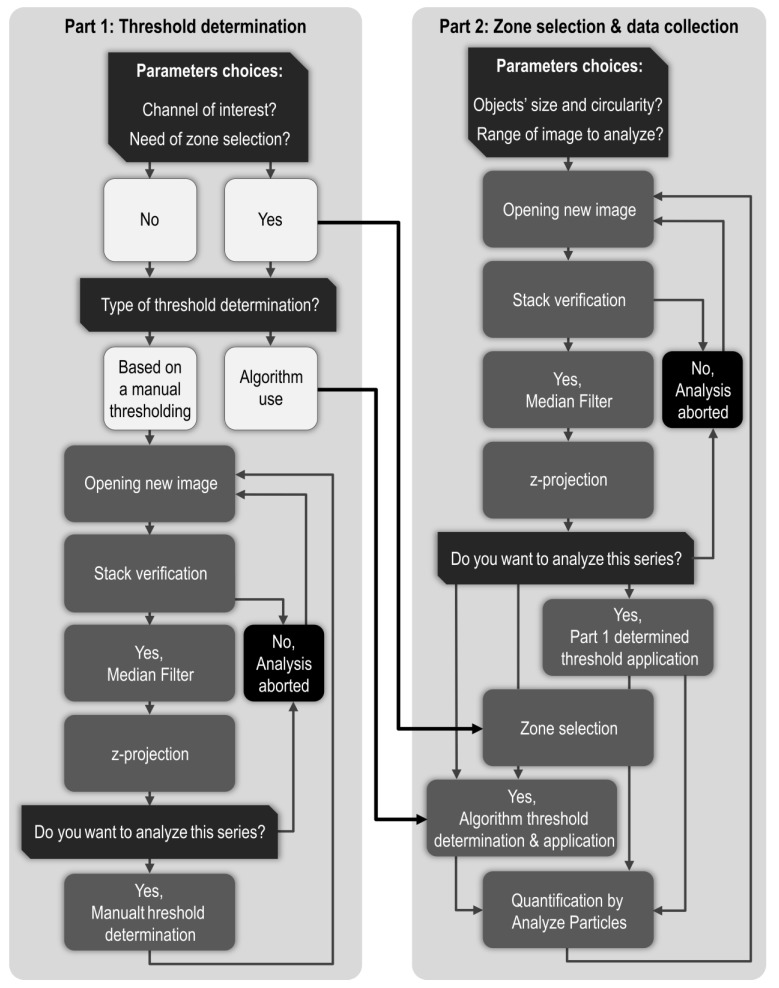
Fiji macro workflow. Giving our image processing protocol, the macro is divided in two major parts. Part 1 (**left panel**) is dedicated to manual threshold determination while Part 2 (**right panel**) is dedicated to zone selection and quantification after application of the previously determined threshold. In Part 1, images are opened, processed according to parameters set in the window presented in Figure 7 and presented to user for threshold determination. Once every image of a .lif file has been treated in Part 1, threshold results are recapitulated before starting Part 2. In Part 2, images are opened again and treated as in Part 1, chosen threshold is applied and the resulting image is presented to user for zone selection before quantification. When every relevant image has been treated, quantification results are superficially analyzed to yield mean, standard deviation, min, and max.

The macro consists of two parts described in Figure 6, the first part allows determination of threshold values, and the second part allows zone selection and quantification.

**Figure 7:**
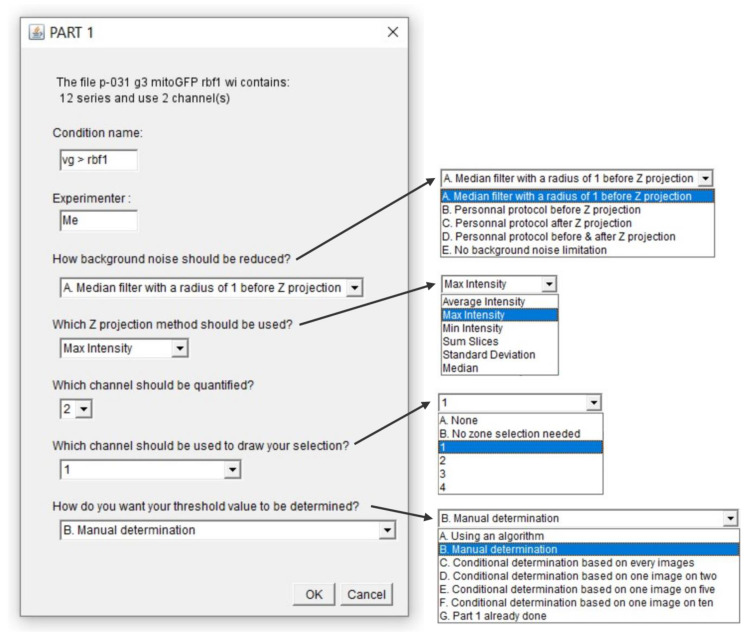
Macro Part 1. The macro starts by collecting few parameters. Importantly, the chosen “Condition name” will end up in the name of the files associated with the .lif file. The default background noise reduction method is a “Median Filter” with a radius of 1 but user can apply its own protocol before and/or after z-projection if needed. As a thresholded image is in black and white, it can hardly be used to define region of interest when a zone selection is needed. Therefore, the macro offers the possibility to define region of interest (i.e., vestigial domain in our case) on another channel or an unthresholded version of channel of interest. If the whole image has to be analyzed, a “No selection needed” option is available. Concerning threshold determination, “A. Using an algorithm” and “B. Manual determination” options will lead to application of an individual threshold value per image. If use of a unique, representative, threshold value to treat every image of a .lif file is wanted, user can make its choice after treating every image (option “C”), 50% of the images (option “D”), 25% of the images (option “E”) or 10% of the images (option “F”). If manual determination has already been carried out, the user may skip Part 1 with option “G”. Once chosen, those parameters are recorded in the Log window which content is ultimately saved in a .txt file.

Once a .lif file has been chosen, the window presented in Figure 7 opens to set a few parameters such as selecting the channels of interest and the methods to use for background noise reduction and z-projection. A more detailed explanation of the macro is provided in the Supplementary Materials.

Once these parameters are set, the macro opens the first image (that should be a stack) and process it according to the chosen parameters for background noise reduction and z-projection. In our case, a “Median Filter” with a radius of 1 is applied and the Max Intensity method is used for z-projection. At this point, threshold can be manually determined on the processed images. Part 1 is over when all images have been processed and as it generates a table of the determined thresholds, Part 2 can be run right after or later.

**Figure 8:**
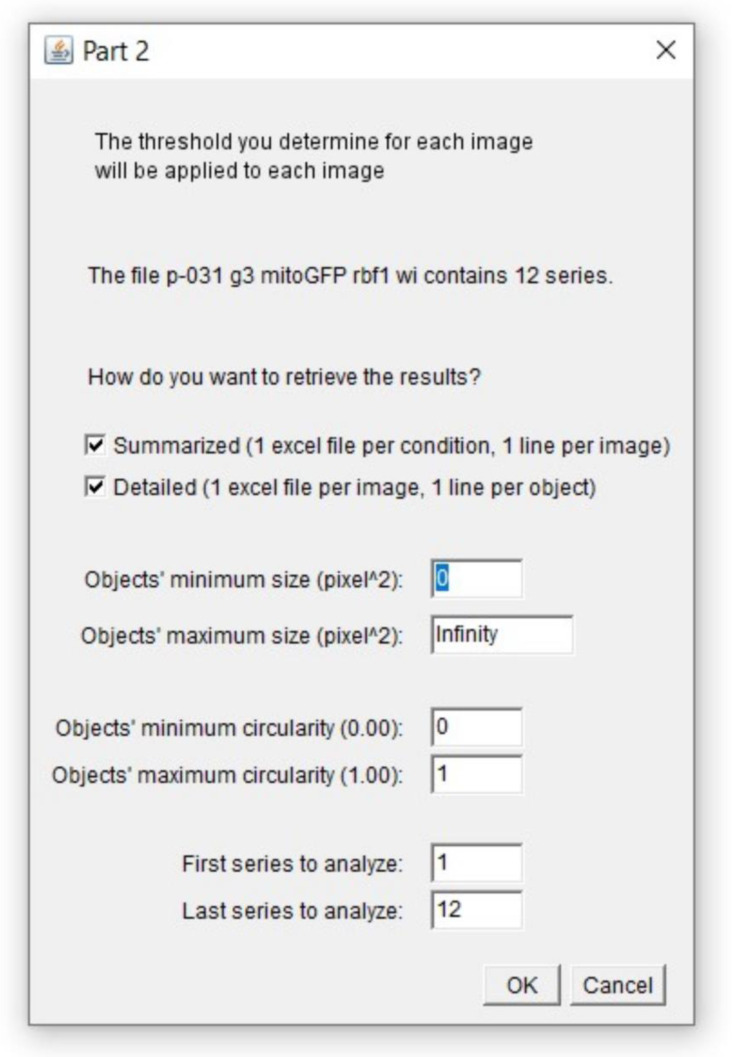
Macro Part 2. Starting window of Part 2 lightly differs according to the choice of threshold method made in Part 1. The one presented here corresponds to option “B. Manual determination”. If option “A. Using an algorithm” was chosen, the window would start by asking which algorithm should be used. If any of options “C”, “D”, “E”, or “F” were chosen, the window would start by asking which unique threshold value should be used. The following parameters (how to retrieve the results, objects’ size, objects’ circularity and range of series to analyze) are always present. Once chosen, those parameters are recorded in the Log window which content is ultimately saved in a .txt file.

Part 2 begins with the opening of the window presented in Figure 8, which offers the possibility to use the threshold value previously determined in Part 1 and to apply additional limitations to quantification such as a size limitation.

Depending on the study, the quantification needed can be more or less detailed. Indeed, one might need to compare objects size within an image whereas in our case the total stained area per image is sufficient. Both options are available on the macro and are not mutually exclusive. Once these settings are carried out, images are processed as in Part 1 except that the chosen threshold is applied. After thresholding, Part 2 will quantify but quantification can be restricted to a region of interest. This region can be drawn on the image of the channel indicated in the Part 1 window. If the thresholding is carried out using an algorithm, the zone selection is carried out before running the algorithm. Indeed, this prevents a highly illuminated artifact outside of the region of interest to weigh in the threshold value determination by the algorithm. To allow a wide range of application for this macro, we have chosen to ask the “Analyze Particles” function to quantify all the possible readouts.

## 4. Conclusions

Apoptosis quantification in a tissue is usually indirect as it generally relies on imaging techniques. There are many ways to analyze an image and once a readout has been chosen, many processing protocols are possible. Here, we describe a semi-automatic protocol running on Fiji for quantification of apoptosis on *Drosophila* wing imaginal discs after TUNEL or activated-caspase labelings. During the development of this protocol, we paid particular attention to the weight of specific steps to obtain a realistic segmentation, which underlies an accurate quantification. As with many image processing protocols, determination of the threshold for binarization turned out to be a critical step. In our case, none of the algorithms we tested was satisfying to determine relevant thresholds.

We also considered using the same threshold value to process several images but, in the end, the best option for our data, was to use a specific threshold manually determined for each image. Indeed, this method proved to carry out a proper segmentation for all images resulting in valid quantification and subsequent detection of biological effects. Even if one could be concerned about the bias that might be induced by this approach, the bias is in fact limited as we observed that threshold values obtained by experimenters are actually highly consistent both for a given experimenter and between experimenters. Moreover, an appropriate processing of the images can facilitate this determination of a threshold value. In this sense, association of a “Median Filter” with a radius of 1 and a Max Intensity z-projection proved to be highly efficient. It would also be interesting to try the “Sum Slices” projection that adds up all pixels’ intensity of a z-axis which should enhance contrast even more (particularly after a median filter) and thus facilitate threshold determination.

The protocol presented here should not affect other readouts available in the “Analyze Particles” function such as: bounding rectangle, shape descriptors, centroid, perimeter, Feret’s diameter, or stack position. Furthermore, we kept the options implemented in CASQITO macro rather open offering a possible use of this tool for a great variety of readouts, stainings, and biological questions.

## Data Availability

The data that support the findings of this study are available from the corresponding authors, I.G. and J.C., upon reasonable request.

## Supplementary Materials

The following are available online at https://www.mdpi.com/article/10.3390/biom11101523/s1, Figure S1: Individual experimenters’ choice of threshold values, Figure S2: Statistical analysis of threshold values consistency, Figure S3: Pairwise comparison of thresholding methods, Supplementary information: User manual of the macro.
