# Supplementary material for: Apoptosis Quantification in Tissue: Development of a Semi-Automatic Protocol and Assessment of Critical Steps of Image Processing
Source: Biomolecules. 2021 Oct 15;11(10):1523. doi: 10.3390/biom11101523 (PMC8533694; doi:10.3390/biom11101523)
Supplement: Supplementary file 1 [file biomolecules-11-01523-s001.zip › biomolecules-1330942-supplementary.pdf]

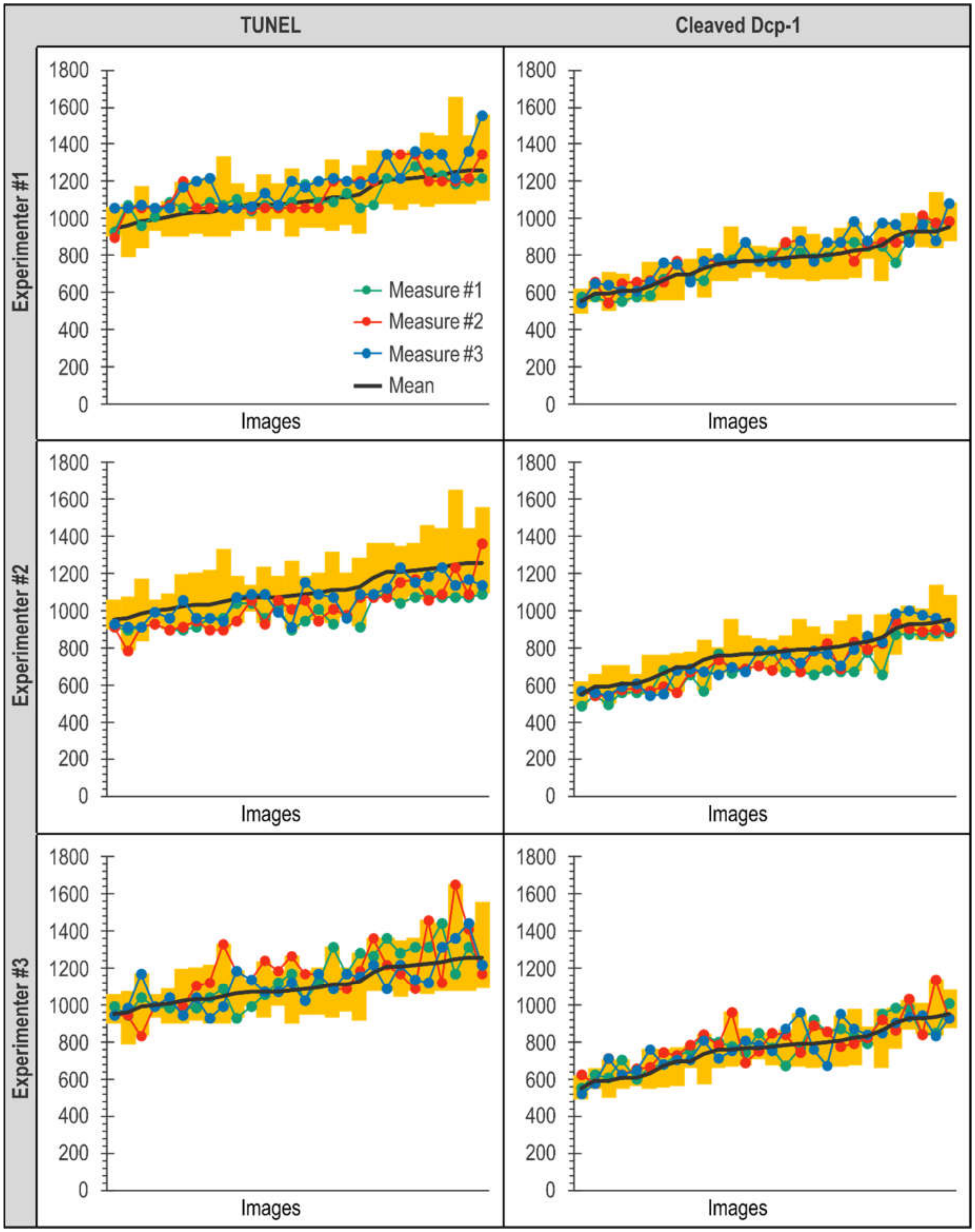

Supplementary Figure S1. Individual experimenters' choice of threshold values.

Results of each experimenter manual determination of threshold in triplicate for the 28 images of the *vg* > *rbf1* genotype are presented for TUNEL (on the left) and anti-cleaved Dcp-1 (on the right). For every experimenter, the first measure is in green, the second in red and the third in blue. In order to facilitate comparison between experimenters, the mean of those 9 measures have been added (in black) and yellow bars correspond to experimenters' range of values for each image.

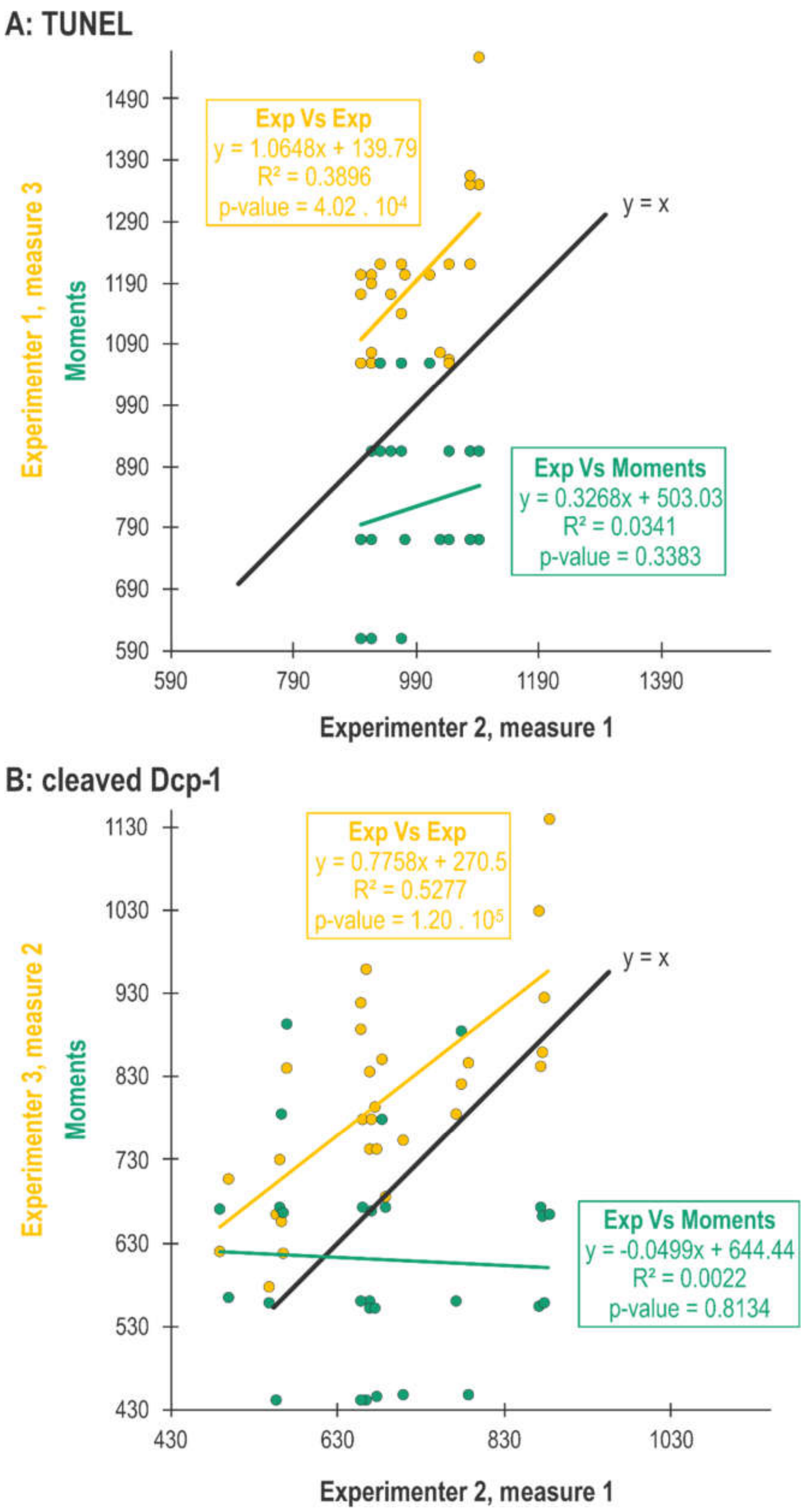

Supplementary Figure S2. Statistical analysis of threshold values consistency

Statistical analysis by linear regression of the threshold values obtained by experimenters or Moments for TUNEL (A) or anti-cleaved Dcp-1 (B) stainings. In yellow, relationship between the two furthest experimenters datasets. In green, relationship between the values obtained with Moments and its closest experimenter dataset.

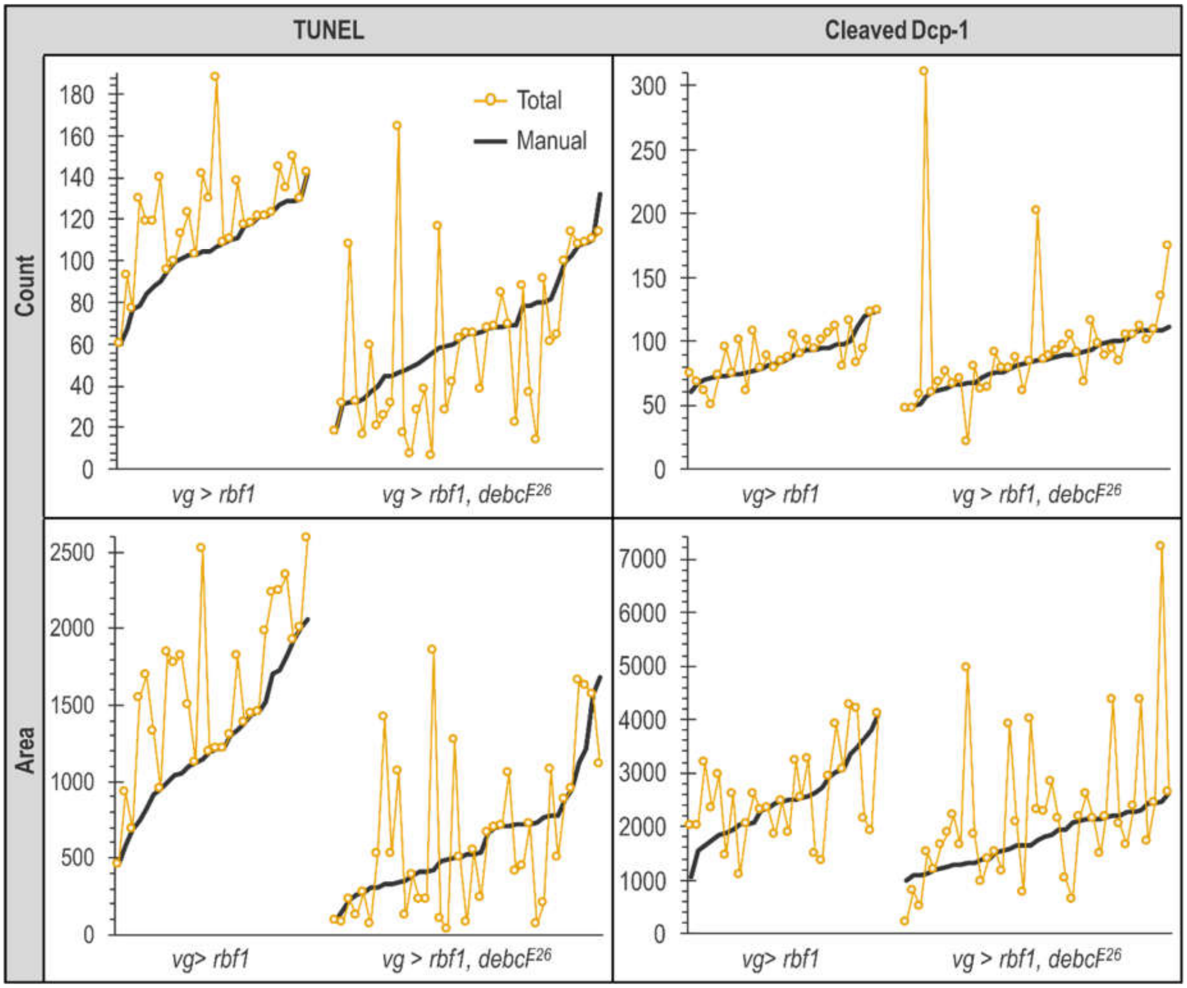

Supplementary Figure S3. Pairwise comparison of thresholding methods

Results presented here are the same as the one presented in Figure 5 (e-h) but in a pairwise organization. Upper panel presents the number of object counted for TUNEL (on the left) and anti-cleaved Dcp-1 (on the right) for both *vg > rbf1* and *vg > rbf1, debcl*^{E26} after applying different thresholding methods (Manual in black, Total in yellow). Bottom panel similarly presents the total number of white pixels after applying different thresholding methods. Images order have been reorganized for each panel in ascending order of results according to Manual method.

Supplementary information: User manual of the macro.

The macro consists of two parts described in Figure 6: the first part allows determination of threshold values and the second part allows zone selection and quantification.

Once a .lif file has been chosen, the window presented in Figure 7 opens to set a few parameters.

These parameters aim at setting the method used for background noise reduction and z projection as well as determining channels of interest. It should be noted that channels are automatically assigned a number that increments in ascending order of the wavelength starting from the shortest wavelength used. Once these parameters are set, the macro opens the first image and checks if it is an actual stack and not a 2D image. In the latter case, the 2D image is skipped without being analyzed and the macro records it has ignored this image in the Log window (“Caution series X is not a stack”) and directly opens next image. When the opened image is a stack, it is processed according to the parameters previously chosen for background noise reduction and z projection. In our case, this means a "Median Filter" with a radius of 1 is applied and z projection is done by the Max Intensity method. Result of this z projection opens twice, giving one image to work on and a second to check in real time if the changes induced are consistent with reality. Just before allowing the user to determine a threshold value, a checkpoint asks for confirmation to treat this image. Indeed, acquisition might have been done on another channel than the one of interest thus, huge artifacts on channel of interest might reveal themselves only at this point. Discarding such image from the analysis is done by clicking the “Do not take series X into account” option. The macro records this image has been ignored in the Log window (“Series X has been excluded from threshold determination”) and directly opens next image. At this step, four windows are open to allow threshold determination: the two images obtained after z projection to monitor the effect of the threshold and two system windows: a “Threshold” window that contains the cursor used to set the threshold value and a "Threshold Selection" window whose “OK” button clicking is required once the threshold has been determined. Importantly, if the “OK” button of the “Threshold Selection” window must be clicked for the macro to continue, buttons of the “Threshold” window should not be clicked. As soon as the “OK” button of the “Threshold Selection” window is clicked, the chosen threshold value is recorded in the Log window and the .xls table and next series is opened. Once all images have been processed, Part 1 is over and a histogram showing the threshold values distribution is displayed. When ready, user have to click the "OK" button of the "End of Part 1" window to start Part 2. Importantly, Part 2 does not necessarily have to be done right after Part 1. As presented in the Part 1 settings choice window (Figure 7), a possible option of the last question is "G. Part 1 already done". This option will lead directly to Part 2. In this case, user can either define a unique threshold value to treat every image of a .lif file or treat each image with its own threshold value after loading the corresponding Excel table containing threshold values for this .lif file obtained in Part 1. This option allows users wanting to apply the same threshold value for all of their conditions to assess the best threshold value for all their conditions in a first time and perform quantification in a second time.

Part 2 begins with the opening of the window presented in Figure 8, which offers the possibility to use the threshold value previously determined in Part 1 and to apply addi-tional limitations to quantification such as a size limitation.

Furthermore, depending on the study, the data needed can be more or less detailed. Here, a global count of the number of objects or total stained area per image was sufficient to be conclusive. Therefore, only a summarized compilation of data was needed. When checked, this option generates an .xls file where each line corresponds to a treated image and its summarized result (i.e. total number of objects or total stained area in the ROI). However, one might need to compare objects size within an image and thus need more detailed data where specifications of every object are recorded. When checked, this option generates an .xls file where each line corresponds to an object (objects of all images are in the same table). Both options are available on the macro and user can choose one or both of them (in which case two .xls files will be generated). Once these settings are done, images are processed as in Part 1 except that, this time, the chosen threshold is applied. After thresholding, Part 2 offers the possibility to limit quantification to a region of interest. If so, the channel needed to draw the selection is opened. Then, when the “OK” button of the “Zone Selection” window is clicked, the macro goes on with quantification and results are recorded in an .xls file. If the settings chosen in Part 1 end up associating a threshold determined by an algorithm with a zone selection, the zone selection is done before running the algorithm. Indeed, to determine a threshold value, algorithms take the whole image into account, which can become a problem if there is a highly illuminated artifact outside of the region of interest. To allow a wide range of application for this macro, we have chosen to ask the "Analyze Particles" function to quantify all the possible readouts.
